# A mixed-methods investigation to understand and improve the scaled-up infection prevention and control in primary care health facilities during the Ebola virus disease epidemic in Sierra Leone

**DOI:** 10.1186/s12889-021-11634-7

**Published:** 2021-08-31

**Authors:** Lara Shiu-yi Ho, Ruwan Ratnayake, Rashid Ansumana, Hannah Brown

**Affiliations:** 1 International Rescue Committee, 1730 M Street NW, Suite 505, Washington, DC 20009 USA; 2 Center for Humanitarian Health, Johns Hopkins University, Baltimore, MD USA; 3 International Rescue Committee, 122 East 42nd Street, New York, NY 10168 USA; 4 Department of Infectious Disease Epidemiology, London School of Hygiene & Tropical Medicine, London, WC1E 7HT UK; 5 Mercy Hospital Research Laboratory, Kulanda Town, Bo, Sierra Leone; 6 School of Community Health Sciences, Njala University, Bo Campus, Sierra Leone; 7 Department of Anthropology, Durham University, Dawson Building, South Road, Durham, DH1 3LE UK

**Keywords:** Ebola, Sierra Leone, Infection prevention and control, Mixed methods research, Humanitarian emergency

## Abstract

**Background:**

The 2014–2015 Ebola epidemic in West Africa became a humanitarian crisis that exposed significant gaps in infection prevention and control (IPC) capacity in primary care facilities in Sierra Leone. Operational partners recognized the national gap and rapidly scaled-up an IPC training and infrastructure package. This prompted us to carry out a mixed-methods research study which aimed to evaluate adherence to IPC practices and understand how to improve IPC at the primary care level, where most cases of Ebola were initially presenting. The study was carried out during the national peak of the epidemic.

**Discussion:**

We successfully carried out a rapid response research study that produced several expected and unexpected findings that were used to guide IPC measures during the epidemic. Although many research challenges were similar to those found when conducting research in low-resource settings, the presence of Ebola added risks to safety and security of data collectors, as well as a need to balance research activities with the imperative of response to a humanitarian crisis. A participatory approach that attempted to unify levels of the response from community upwards helped overcome the risk of lack of trust in an environment where Ebola had damaged relations between communities and the health system.

**Conclusion:**

In the context of a national epidemic, research needs to be focused, appropriately resourced, and responsive to needs. The partnership between local academics and a humanitarian organization helped facilitate access to study sites and approvals that allowed the research to be carried out quickly and safely, and for findings to be shared in response forums with the best chance of being taken up in real-time.

## Background

### Humanitarian context

Infection prevention and control (IPC) in primary health care facilities and hospitals in developing countries is known to be wholly inadequate with the recorded incidence of healthcare-acquired infection being three-fold higher than that recorded in the United States [1]. Poor IPC has served as an efficient amplifier of transmission of viral hemorrhagic fevers including Ebola and Lassa [2, 3]. Studies have shown that in conflict-affected settings where these outbreaks occur, including the Democratic Republic of Congo (DRC), Uganda, and Angola, IPC infrastructure, investment, and training in IPC are poor [2–5].

During the 2014–2015 Ebola virus disease (EVD) epidemic in West Africa, lack of adequate IPC practice and materials in primary health care facilities was a main driver of health care worker (HCW) infections and subsequently, community transmission. The vast majority (66%) of HCW infections occurred in primary health units and hospitals [6] and was related to poor IPC, which included at various points during the epidemic, a lack of available IPC materials, a lack of familiarity with principles of IPC, and inexperience in the safe use of IPC [7]. Thus it is conceivable that poor IPC may amplify nosocomial, and then community transmission of viral hemorrhagic fevers (VHF), as has been seen in previous VHF outbreaks. The epidemic overwhelmed a health system that was still recovering from a brutal civil war (1991–2002), and which also suffered from extensive health system fragmentation, high levels of external dependence, and poorly-coordinated policy development [8]. The under-five mortality rate at the time was among the highest in the world and ranked under only Angola, Chad, Somalia, and the Central African Republic [9]. Neighboring Liberia was also recovering from two civil wars (1989–1997 and 1999–2003). Together, these weak health systems were unable to cope with the epidemic, leading to a humanitarian crisis across the region’s porous borders. Gaps in IPC were clear from the incidence of EVD among HCWs, which reached 100 times that of the general population, leading to the death of nearly 10% of the workforce [6].

## Main text

### Research study

Conducted during the peak of the EVD epidemic in late 2014 in Sierra Leone, the primary objective of the study was to rapidly generate insights on how IPC behaviors of HCWs at primary healthcare (PHC) facilities could be rapidly improved during an EVD outbreak. A secondary objective was to understand the perspectives of health facility staff and health committees (elected community representatives) on the use of PPE, in order to improve knowledge and acceptance among HCWs and the communities they serve. This would inform ongoing efforts by the Ebola Response Consortium (ERC), a group of non-governmental organizations led by the International Rescue Committee (IRC), to support HCWs in scaling-up IPC in 1200 primary health units (PHUs) across the country as quickly and effectively as possible. The IRC received a rapid response grant from the Research for Health in Humanitarian Crises (R2HC) programme to directly observe and evaluate adherence to IPC, discuss in detail HCW attitudes and experiences with IPC, and work with HCWs to improve practices in real-time.

Early on in the epidemic, the response emphasized IPC in hospitals where most of the suspected EVD cases were being isolated before they could be taken to Ebola treatment units. However, the point of contact with the health system often remained the initial PHU providing basic health services to their local community. HCWs at these facilities faced daunting logistical and personal challenges to their determination to protect themselves and their patients and stigmatization, while providing life-saving maternal, newborn, and child health care that was frequently sidelined by the needs of the widespread epidemic. Moreover, safe use of the sometimes dramatically imposing personal protective equipment (PPE) sometimes caused fear in locals and physically isolated HCWs from patients, thus dissuading community-based care [2, 7, 10].

A single-group, pretest-posttest design was employed, which used a participatory action framework and a mixed-methods approach to evaluate the effects of a post-baseline workshop to develop and execute improvement plans for IPC tailored to each facility. During the peak in the national caseload in December 2014, more than 400 confirmed cases were reported weekly [11]. We conducted the study from December 2014 to January 2015 in Bo and Kenema districts within eight PHUs that provided primary healthcare services. The Ministry of Health and Sanitation (MoHS) and the Ebola Response Consortium led the national IPC trainings for HCWs, which were completed approximately one week before data collection began.

We used both quantitative and qualitative methods at baseline and follow-up. This included in-depth interviews with HCWs and focus group discussions with health management committee members, and self-administered surveys for HCWs to examine attitudes and self-efficacy towards IPC. In addition, we measured the adherence to IPC among HCWs via structured observations of their actual behaviors with all patients presenting to the facility at the baseline and follow-up period. Changes in the proportion of behaviors adherent to IPC were evaluated statistically, based on the protocol health workers had been trained on. The intervention involved workshops for District Health Management Teams (MoHS staff at the district level responsible for oversight of the health activities), HCWs, and community members to develop improvement plans for their own facilities. This included a process of generating and implementing ideas in response to their biggest challenges in dealing with IPC.

Although there were no changes to the overall study design, a trial phase of the improved IPC practices could not be applied to assess the feasibility of improving specific practices. This was due to the plans developed by workshop participants being more general than was intended and lack of ability of investigators to follow up closely in person due to the rapidly evolving outbreak. The improvement plans therefore were carried out in each facility, but not always on the intended timeline of three weeks due to the competing priorities to support the epidemic response.

The results have been explored in three papers [12–14]. There was an overwhelming conviction among HCWs that IPC is lifesaving, which helped HCWs overcome the strong physical discomfort and sense of distance with patients that it caused [13]. The improvement plans focused on core elements: improving the screening of patients before they came into the facility; maintaining physical distance; and reinforcing patient handwashing. It follows that statistically significant changes in these domains were observed at follow-up. However, handwashing among HCWs, glove-changing between patients, and detailed questioning for symptoms and risk factors were poorly adhered to across rounds of data collection.

Interviews highlighted the psychosocial impacts of the outbreak on healthcare workers, and focus group discussions demonstrated how community members participated in the response. Concerning IPC specifically, HCWs described the challenges of using PPE despite understanding its lifesaving qualities [14]. We identified three main hypotheses about HCWs’ adherence to IPC protocols relating to rational thinking, individual risk determination, and peer bonding vs. self-protection that can inform how to address challenges in improving IPC adherence in primary healthcare facilities. Finally, we detailed the roles, responsibilities, and concerns of community volunteers and health management committees in addressing the epidemic at the often-forgotten local level.

### Scientific importance

The study identified ample gaps in the implementation of IPC during an epidemic and humanitarian crisis. This provided the rationale to improve current training packages that focus on physical structures and knowledge, including contextual, emotional, psychological, and behavioral factors that also influence adherence to IPC practice and the motivations of HCWs. An active epidemic presents serious challenges to conducting operational research but a crucial opportunity to examine actual HCW behaviors and attitudes under duress, and can inform policy and practice. This was a rare opportunity that added to the knowledge gained from previous epidemics in DRC, Uganda, and Angola.

Although disease outbreaks are more likely to happen in fragile settings where health systems are weak, they can occur anywhere, and preparedness is key to ensuring they do not spread. Learning from this case can be applied to IPC preparedness in any setting. As evidenced by the prolonged EVD outbreak in North Kivu and Ituri Provinces of the DRC, which started in 2018 and is now the second largest outbreak in history, EVD continues to be a disease of global public health concern. Prevention and control require re-writing standards for preventative IPC across all health care facilities and during the epidemic, motivated, trained, and equipped HCWs to continue to drive containment for a sustained period of time. If anything, we can expect to see growing numbers, scale, and duration of Ebola outbreaks in the future. Their occurrence in humanitarian settings simply makes them more challenging to contain, but the recent cases imported from DRC into Uganda highlight both that population movements from crisis settings will not limit transmission to the original setting and that a strong infection control approach in Uganda can indeed promote rapid containment [15].

We planned a practical dissemination strategy that reflected the nature of research during a current outbreak response. First, stemming from the participatory nature of the study, during the study period itself, we attempted to carry out improvements to the physical infrastructure that HCWs had voiced as being essential, as these were integral to improving IPC behaviours at the health facility level. Second, we emphasized rapid sharing of the main results with operational partners who could act on the findings: the District Health Management Teams, our partners in the Ebola Response Consortium (e.g., Action Contre La Faim, Save the Children, ABC Development, etc.), national MoHS officials, and the IPC-focused sub-group of the national Ebola response. Third, we developed technical documents and presentations for Sierra Leone’s national biomedical scientific conference, and we rapidly published three peer-reviewed papers, a policy brief on psychosocial impacts of the outbreak, and presented the findings at the 2015 American Society for Tropical Medicine and Hygiene Annual Meetings and 10th European Congress on Tropical Medicine. The findings on psychological stress of HCWs helped prompt and inform an IRC developed self-care intervention for HCWs post-Ebola [16].

At a micro-level, we implemented the study successfully with great enthusiasm by HCWs and District Health Management Teams and allowed for actual implementation of improvement plans. At the macro-level, that Sierra Leone’s National Recovery Plan for 2015–2017 put $33 million towards scaling up and maintaining IPC in both PHUs and Ebola treatment units, in order to prevent a recurrence of EVD, likely reflects the combined contributions of real-time research and evaluation done through collaboration between the MOHS, IRC, UNICEF and the US CDC across Sierra Leone [17, 18]. IPC was also added to university curriculum at Njala University and University of Sierra Leone [19, 20]. Organizationally, strengthening IPC has been the focus and core of the IRC’s subsequent EVD responses in Equateur and North Kivu provinces of DRC in 2018 and 2019, and its work in EVD preparedness in Uganda.

### Challenges to research in a unique humanitarian setting

Due to the complex technical nature of disease control for EVD and the sheer magnitude and geographical distribution of the epidemic, the EVD response in West Africa mirrored that of a classical humanitarian response which required speed and scale that far outstripped the capacity of the countries affected [21]. Therefore, conducting research and evaluation in real-time to aid the response presented challenges exacerbated by the crisis context and the required multisectoral response, but also the risks of EVD for HCWs and researchers alike.

#### Balancing research with emergency response

Concurrent to the research, the IRC was mounting a large-scale emergency response to the EVD outbreak, which meant it was imperative that the IRC health program staff be physically and mentally focused on the emergency response. Therefore, they had limited time to spend supporting research in the way they normally would have in the absence of acute crises (for example, participating in research design and data collection, giving advice on contextual issues, etc.). Co-investigators were not able to spend as much time in the field as would have been ideal because of competing responsibilities related to the emergency response. In addition, transportation and other shared resources were more limited than usual because they were needed for the response. This scenario required that the research design be tailored to the acute needs of the program in improving IPC practice, and be responsive to the program in requiring minimal physical and humanitarian resources to execute in a timely fashion.

#### Ensuring access and local expertise

The crisis itself caused trust issues between communities, HCWs, authorities, and other responders, so it was essential to build a competent team both with recognized EVD experts who knew the content well from previous outbreaks, but also those who could travel to study sites and move freely across the country [22]. Therefore, contrary to the dominant scenario in global health where global academic experts lead the design and implementation of the study, we shifted to a model where the IRC research advisors, Sierra Leonean advisors, and where feasible, the country team, actively took the lead in designing feasible study designs and led the implementation, with expertise on EVD delivered largely remotely from global academic experts. However, in retrospect additional staffing to follow the study activities full-time over the entire period would have been beneficial as the usual reliance of program staff to share some responsibilities was not realistic in this situation.

#### Safety and security of researchers and staff

Given its severity and potential political unrest and community resistance, we had to presume that EVD research posed risks to field researchers more akin to conducting research in an active conflict setting rather than a regular outbreak situation. Therefore, the IRC as an organization had to ensure safety from infection and protection both from EVD and stigma for Sierra Leonean and international investigators and data collectors who would be visiting health facilities. This required internal discussions with IRC’s legal counsel and human resources to ensure research staff were protected by the same procedures (i.e., security, debriefing, evacuation, etc.) in place for IRC staff who were responding to the outbreak, before requesting research funding and securing commitments from the institutions involved. In retrospect, these risks did not emerge, but this scenario demonstrated the cascade of considerations that must be in place a priori to engage in cross-institution field research during a severe epidemic.

### Research strategies to address challenges

#### Participatory approach

Given the trust issues that were apparent from the start of the outbreak between citizens, health systems, and the response, we chose an explicitly participatory approach. The IRC had existing relationships with the Bo and Kenema District Health Management Teams, as did one of the co-investigators, and they were engaged in discussions about study design during proposal development and before the start of data collection. In turn, the District Health Management Teams engaged the selected health facility staff to reach out to community health workers and health committee members. These community level structures were already supported by IRC health programming. Such pre-existing relationships were important in gaining trust and being able to move the research forward in a short timeframe. The IRC’s work with the MoHS at the central level meant that we had existing lists of all health facilities and were able to stratify and sample facilities early on. Healthcare workers, community members involved in baseline interviews and surveys, as well as District Health Management Team staff were invited to a workshop to review and analyze the preliminary results, and develop action plans to trials of improved practices to address gaps identified at baseline.

#### Partnership between local academics and humanitarian organizations

We contacted investigators involved in an ongoing research collaboration on Lassa fever between Mercy Hospital Research Laboratory in Sierra Leone, Durham University in the United Kingdom, and Charité – Universitätsmedizin in Germany. Together, we decided that the Sierra Leonean investigators with the most familiarity with the national research apparatus should take the lead role in laying the groundwork for the study, including the institutional review board (IRB) approval process and preparing the fieldwork with IRC, in the midst of the crisis. This was key for advancing the study while national institutions were less functional and where protocol development had to be realistic and accelerated with regard to the short timeline. Given the screening and quarantine issues upon return, some universities in high income countries were not allowing their staff to travel to affected countries independently to conduct research. Therefore, the IRC made the decision early on to lead the study internally with principal investigators and collaborators already in-country to support the response and deliver expertise on IPC who were able to work with the local academic to recruit, train, and supervise the data collection team. The one academic investigator who was able to travel was then supported by the IRC when she went to Sierra Leone.

#### Rapid and focused response

With limited time to train staff, we hired data collectors who had previously worked with Mercy Hospital in Bo or IRC. Finally, other expertise was brought on as needed, including a qualitative researcher to drive the analysis of the large amount of qualitative data. As a team, we made rational and feasible modifications to the protocol and data collection in the field, in accordance with the ethical profile outlined under the IRB approval.

## Conclusions

A major lesson learned was that rapid response research during a severe epidemic should have very specific aims and methods that can be quickly understood and efficiently delivered without detracting from the emergency response. We achieved this for the most part but could have been more realistic about the scale of the intervention (improvement to IPC) that would be possible in a limited timeframe with many competing priorities. This is detailed in the main study publication [12].

We also learned that the qualitative insights from HCWs interviews were rich, crucial, and entirely unexplored. This led to two articles that shed light on first, the psychosocial effects of the live-saving IPC and Ebola management painstakingly carried out by dedicated HCWs in communities, and second, the crucial and less-discussed interface between communities and health facilities in supporting Ebola prevention and control [13, 14].

This was an unprecedented type of crisis, and hence, it produced a unique case study. The humanitarian enterprise had never before experienced an outbreak of this scale and severity, and real-time research was essential to finding new solutions to address the emergency now and inevitably, in the future. The engagement of both a humanitarian organization that needed answers and local academics who knew the terrain best facilitated the ability to carry out research under these conditions, and as the funder intended, increased the likelihood of uptake of the results. The existing relationships that these parties had were essential to being able to carry out the research in the midst of the epidemic. That context also made it necessary to take precautions beyond the normal measures to ensure the safety of not only the research subjects, but the research staff. The IRC’s policies and procedures for staff safety in outbreaks have evolved and been further institutionalized during subsequent Ebola outbreaks and the current COVID-19 pandemic. At the same time, the epidemic made it clear that dedicated human and financial resources for research must be available in a crisis because existing program staff cannot be expected to engage in the way they might under non-emergency conditions. Had there been more time for investigators or a dedicated research manager to conduct follow up visits after the workshops to monitor the implementation plans we may have seen more fidelity to them. This experience has informed the design of a study financed by the same donor for the current EVD crisis in Uganda, primarily in terms of more adequately resourcing the research team on the ground given concurrent response activities and engaging local researchers.

## Data Availability

Not applicable.

## Abbreviations

EVD: Ebola Virus Disease
HCW: Healthcare workers
IPC: Infection Prevention and Control
IRB: Institutional Review Board
IRC: International Rescue Committee
MoHS: Ministry of Health and Sanitation
PHU: Peripheral Health Unit
PPE: Personal protective equipment
WHO: World Health Organization
